# Family history of type 2 diabetes and the risk of type 2 diabetes among young and middle‐aged adults

**DOI:** 10.1002/cdt3.147

**Published:** 2024-07-23

**Authors:** Ken R. Smith, Huong Meeks, David Curtis, Barbara B. Brown, Kyle Kole, Lori Kowaleski‐Jones

**Affiliations:** ^1^ Department of Family and Consumer Studies University of Utah Salt Lake City Utah USA; ^2^ Department of Pediatrics University of Utah Salt Lake City Utah USA

**Keywords:** epidemiology, family history, family studies, obesity, type 2 diabetes

## Abstract

**Background:**

The prevalence of type 2 diabetes has been growing among younger and middle‐aged adults in the United States. A portion of this increase for this age group may be attributable to shared type 2 diabetes risks with family members. How family history of type 2 diabetes history is associated with type 2 diabetes risk among younger and middle‐aged adults is not well understood.

**Methods:**

This population‐based retrospective cohort study uses administrative, genealogical, and electronic medical records from the Utah Population Database. The study population comprises offspring born between 1970 and 1990 and living in the four urban Utah counties in the United States between 1990 and 2015. The sample comprises 360,907 individuals without a type 2 diabetes diagnosis and 14,817 with a diagnosis. Using multivariate logistic regressions, we estimate the relative risk (RR) of type 2 diabetes associated with the number of affected first‐ (FDRs), second‐ (SDRs), and third‐degree (first cousin) relatives for the full sample and for Hispanic‐specific and sex‐specific subsets.

**Results:**

Individuals with 2+ FDRs with type 2 diabetes have a significant risk of type 2 diabetes in relation to those with no affected FDRs (RR = 3.31 [3.16, 3.48]). Individuals with 2+ versus no SDRs with type 2 diabetes have significant but lower risks (RR = 1.32 [1.25, 1.39]). Those with 2+ versus no affected first cousins have a similarly low risk (RR = 1.28 [1.21, 1.35]). Larger RRs are experienced by males (2+ vs. 0 FDRs, RR = 3.55) than females (2+ vs. 0 FDRs, RR = 3.18) (*p* < 0.05 for the interaction). These familial associations are partly mediated by the individual's own obesity.

**Conclusions:**

The risks of type 2 diabetes are significantly associated with having affected first‐, second‐, and third‐degree relatives, especially for men. One of the forces contributing to the rising patterns of type 2 diabetes among young and middle‐aged adults is their connection to affected, often older, kin.

## Highlights

- Rates of type 2 diabetes are increasing among younger and middle‐aged adults.
- Among young and middle aged‐adults, the risk of type 2 diabetes increases for individuals with a family history of type 2 diabetes, especially when there are more affected relatives, particularly, parents and siblings.
- More distant kin, including aunts, uncles, grandparents, and cousins who are affected also increase this risk.
- The risk attributable to family history is greater for men than women though for both sexes; the association is moderated by whether the individual is obese.
- Enhancing awareness of the risk of a family history of diabetes among younger adults could help target preventive efforts.

## Graphical abstract

Young and middle‐aged adult's excess type 2 diabetes risk is associated with strong family history of type 2 diabetes, especially for men.

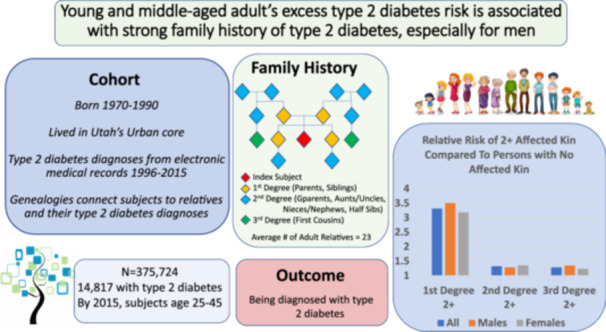

## 1 INTRODUCTION

Type 2 diabetes is a significant public health problem in terms of its association with disability and mortality affecting a large fraction of the US population, though historically concentrated among older adults.^1^, ^2^, ^3^, ^4^ There is growing concern over the increasing prevalence among younger and middle‐age adults in the United States^1^ with three‐quarters of children with type 2 diabetes having an affected close relative.^4^ The objective of this paper is to examine the risk of type 2 diabetes among young and middle‐aged adults and how this risk is related to family history of type 2 diabetes in the context of rising rates of diabetes.

The prevalence of type 2 diabetes has been rising for children up through middle‐aged adults.^5^, ^6^, ^7^, ^8^ The incidence of type 2 diabetes in younger persons may be connected to the risks encountered by relatives over age 45.^9^, ^10^ In general, adults across all ages with a family diabetes history are at an increased risk of type 2 diabetes themselves,^11^, ^12^, ^13^, ^14^, ^15^, ^16^, ^17^, ^18^, ^19^ and the risk declines as the affected relatives moves from close to more distant relatives.^19^ Recent evidence that identifies the genetic contributions to familial risk exists, although the contribution of the shared family environment remains unclear.^20^, ^21^

The risks attributable to a family diabetes history may not be uniform across different key subpopulations. A growing but somewhat inconsistent body of evidence shows how familial or genetic risk of type 2 diabetes varies by sex,^17^, ^22^ race, and ethnicity.^23^, ^24^, ^25^

In this study, we add new insights about the role of familial risk among young and middle‐age adults. This is done in several ways. First, like Weires et al.,^19^ the role of first‐ (FDRs), second‐ (SDRs), and third‐degree relatives is assessed without requiring that subjects are embedded in a three‐generation pedigree. Second, statistical controls for the number of relatives in the proband's kindred are introduced to better account for variation in pedigree size since a family history of a disease is more likely to be observed if there are more kin. Third, this study includes another two decades of data beyond the Weires et al. analysis^19^ during which time both type 2 diabetes and obesity rates have increased. Fourth, the potential mediating influences of an individual's body mass index (BMI) and co‐morbidities are examined. Fifth, the differential effects of family diabetes history by ethnicity and sex are addressed. In sum, we consider the risk of type 2 diabetes among young and middle‐aged adults in terms of their family diabetes history.

## 2 METHODS

### 2.1 Data source

This study is based on data drawn from the Utah Population Database (UPDB).^26^, ^27^ The UPDB is one of the world's richest sources of linked population‐based information for demographic, genetic, and epidemiological studies at the individual level, drawing from statewide records for all‐payer (third party) claims data (APCD), vital records (i.e., birth and death certificates), health facilities data (i.e., inpatient hospitalizations, ambulatory surgeries, emergency department visits), and driver licenses. Selection bias is minimal given the population coverage of UPDB.

UPDB data permit investigators to use genealogical information that connect individuals with their relatives (all anonymized). To study the familial recurrence of any health condition, this feature is essential and makes the UPDB ideal for this study.

### 2.2 Sample selection

The specific UPDB information needed for this analysis spans 1950–2015. To ascertain a diabetes diagnosis, APCD data (2008–2019), death certificates (1990–2019), and healthcare facilities data (1996–2019) were used. Those identified as being diagnosed with type 2 diabetes have International Classification of Diseases (ICD) Revision 9 codes of 250.X0 and 250.X2 or ICD10 codes of E08 or E11. Using consistent and comprehensive medical records serves to minimize reporting biases associated with self‐reported medical conditions.

Eligible persons comprise individuals born between 1970 and 1990 with parents born between 1950 and 1970. Individuals also resided in one of the four Utah urban counties (i.e., Salt Lake, Davis, Utah, or Weber counties) between January 1990 and December 2015. Accordingly, the sample includes people between the ages of 25 and 45 by 2015. The relatives of this sample who serve as the basis for measuring family diabetes history are FDRs (parents and siblings), SDRs (grandparents, uncles, aunts, nephews, nieces, and half‐siblings), and third‐degree relatives (exclusively first cousins, FCs).

The offspring cohort that met the birth date and residential history requirements numbered 421,841. Individuals (offspring and their parents) were also required to have lived in Utah between 1996 and 2019 after age 18. This period covered the years when statewide medical records are available to ascertain a type 2 diabetes diagnosis. This meant that 28,542 offspring whose parents did not meet this latter requirement were removed along with 17,979 offspring who themselves did not satisfy this eligibility rule. Finally, 46 offspring were removed where the recorded sex of the parent in UPDB was inconsistently recorded (e.g., mother's sex was male).

The resulting sample totals an *N* = 375,724 comprising 360,907 and 14,817 without and with type 2 diabetes, respectively.

### 2.3 Statistical analysis

To generate estimates of risk, the statistical strategy involves the estimation of multivariate logistic regressions where the dependent variable is whether or not the individual has been diagnosed with type 2 diabetes. The key independent variables comprise indicators of family type 2 diabetes history where several alternative specifications and operationalizations were adopted. All models adjust for the number of eligible relatives for a given degree of genetic relationship for whom their diabetes status could be determined. To enhance the basis for supporting causal associations, the diagnoses of relatives are largely diagnosed at ages before the diagnoses of the subjects, and all confounders are fixed (exogenous) covariates. All analyses were conducted using SAS 9.4.^28^

Given differences in the prevalence of type 2 diabetes in our young sample by sex and by Hispanic status, sex‐specific and Hispanic status‐specific models are estimated. In this study, females have higher prevalence rates than males (4.6% vs. 3.3%) and Hispanic individuals have higher rates than non‐Hispanic persons (6.4% vs. 4.1%). For the full sample, models are estimated that allow for effect modification of family diabetes history by Hispanic status and by sex.

### 2.4 Measures of family history

The models estimated include indicators of family diabetes history for FDRs, SDRs, and FCs concurrently. To address the distinct effects of each type of relationship, additional models are estimated to show how each of the three family‐relation types are associated with type 2 diabetes when included one at a time.

Several indicators of family history of type 2 diabetes were applied similarly to FDRs, SDRs, and FCs: (1) whether there are any affected eligible relatives (dummy variables); (2) number of affected relatives (integer count); (3) number of affected relatives (categorical: 0, 1, 2+); (4) proportion of relatives who were affected (continuous); and (5) proportion of relatives who were affected (categorical: quartiles).

Each of these distinct operationalizations are used to assess the sensitivity of our assessment of familial diabetes risk. While results varied somewhat, they agree with respect to how family diabetes history is associated with an individual's risk of type 2 diabetes. The results for specification #3 are highlighted here because it easily conveys the general risk profile as well as the nonlinear pattern of the associations.

### 2.5 Confounder and mediating variables

In addition to our measures of family history (number of affected relatives and number of eligible relatives), all models include covariates such as the individual's sex, birth year, whether non‐White race, whether Hispanic ethnicity, and level of parents' maximum education. Parents' education is used rather than the individual's own education because the UPDB obtains information about the education level from statewide birth certificates. Accordingly, Utah‐born individuals all have parental education data. Both father's and mother's education are considered, but only the greater of the two is included. The individual's own education level is observed only if they themselves became parents in Utah, which would have reduced the sample size. For variables with missing data, a commensurate “missing” dummy variable is included.

The potentially strong mediating effects of health status and BMI are addressed in this analysis. The Charlson Comorbidity Index (CCI)^29^, ^30^ is a long‐standing and validated indicator of health status; here, this index is used but excludes diabetes as a component of the measure. The full CCI includes two levels of diabetes: with and without complications. Both of these are excluded in the CCI variable used here. BMI was included as a binary variable representing individuals who were obese (BMI ≥ 30) or not by age 30, where BMI was derived from driver license data that has been shown to measure obesity reliably.^31^

A set of four models (A–D) are estimated where the logistic regressions included the family history variables and the aforementioned covariates (Model A) excluding CCI and obesity status. This is followed by models that add the CCI (Model B) and then models that excluded the CCI but included our obesity dummy variable (Model C) and then finally one that included both the CCI and obesity dummy concurrently (Model D).

## 3 RESULTS

### 3.1 Descriptive statistics

In our sample, those with type 2 diabetes are more likely to be female, slightly older, less likely to be White, more likely to be Hispanic ethnicity, born to parents with lower levels of education, and more likely to be obese (Table 1). Descriptive statistics are provided regarding the composition of relatives, the number that are eligible for detecting diabetes in their Utah medical records, and the distribution of their diagnosis status. In general, those diagnosed with type 2 diabetes are more likely to have higher numbers of affected kin in relation to those who have not been diagnosed.

**Table 1:** Demographic characteristics of the study cohort by type 2 diabetes status.

Characteristics	No type 2 diabetes, *N *= 360,907	Yes type 2 diabetes, *N *= 14,817
	*N* (%) or mean (SD)	*N* (%) or mean (SD)
Sex		
Female	175,511 (48.6%)	8530 (57.6%)
Male	185,396 (51.4%)	6287 (42.4%)
Birth year	1982.5 (5.0)	1980.2 (5.2)
National Institutes of Health race categories		
White	343,483 (95.2%)	13,602 (91.8%)
American Indian or Alaska Native	758 (0.2%)	67 (0.5%)
Asian	1215 (0.3%)	27 (0.2%)
Native Hawaiian/Pacific Islander	791 (0.2%)	76 (0.5%)
Black or African American	919 (0.3%)	56 (0.4%)
Multiple races	12,153 (3.4%)	967 (6.5%)
Unknown	551 (0.2%)	6 (0.0%)
Presumed not White, cannot be classified	1037 (0.3%)	16 (0.1%)
Whether Hispanic		
No	287,535 (79.7%)	12,318 (83.1%)
Yes	31,020 (8.6%)	2106 (14.2%)
Unknown	42,352 (11.7%)	393 (2.7%)
Maximum parental education level		
Less than HS	9232 (2.6%)	765 (5.2%)
HS degree	74,999 (20.8%)	4161 (28.1%)
Some college	114,597 (31.8%)	4981 (33.6%)
College degree	68,778 (19.1%)	2198 (14.8%)
Postcollege	91,591 (25.4%)	2643 (17.8%)
Unknown	1710 (0.5%)	69 (0.5%)
Obese before age 30 (BMI)		
Yes	49,162 (13.6%)	5796 (39.1%)
No	285,363 (79.1%)	6848 (46.2%)
Unknown	26,382 (7.3%)	2173 (14.7%)
Maximum CCI excluding diabetes before age 30		
0	349,829 (96.9%)	14,424 (97.3%)
1	6295 (1.7%)	244 (1.6%)
2	1822 (0.5%)	71 (0.5%)
3+	2956 (0.8%)	78 (0.5%)
Number of eligible FDRs^a^	4.1 (2.0)	3.7 (1.9)
Number of eligible FDRs with type 2 diabetes		
0	233,475 (64.7%)	5977 (40.3%)
1	100,924 (28.0%)	5663 (38.2%)
2+	26,508 (7.3%)	3177 (21.4%)
Number of eligible SDRs^b^	7.9 (6.1)	8.1 (5.2)
Number of eligible SDRs with type 2 diabetes		
0	79,268 (22.0%)	2660 (18.0%)
1	82,174 (22.8%)	2889 (19.5%)
2+	166,506 (46.1%)	8220 (55.5%)
No eligible relatives	32,959 (9.1%)	1048 (7.1%)
Number of eligible FCs^c^	11.2 (11.7)	10.7 (10.8)
Number of eligible FCs with type 2 diabetes		
0	185,564 (51.4%)	6599 (44.5%)
1	62,406 (17.3%)	2785 (18.8%)
2+	37,531 (10.4%)	2381 (16.1%)
No eligible relatives	75,406 (20.9%)	3052 (20.6%)

Abbreviations: BMI, body mass index; CCI, Charlson Comorbidity Index; FC, first cousin; FDR, first‐degree relative; HS, high school; SDR, second‐degree relative.

^a^ FDRs, which include parents and siblings.

^b^ SDRs, which include aunts, uncles, grandparents, nieces, nephews, and half siblings.

^c^ FCs, which represent a portion of third‐degree relatives.

### 3.2 Logistic regressions: Main effects of family history estimated concurrently

In models where all family history variables are included concurrently (Model A) (Table 2), individuals with a single affected FDR have nearly a two‐fold increase in the risk of type 2 diabetes (relative risk [RR] = 1.98, 95% confidence interval [CI]: 1.91, 2.06), and this risk increases to more than four‐fold (RR = 4.24; 95% CI: 4.04, 4.44) when there are two or more affected relatives. Risk estimates for having a single or 2+ affected SDR and FC are practically identical: one affected relative: RR (SDR) = 1.10, RR (FC) = 1.10; 2+ affected relatives: RR (SDR) = 1.36, RR (FC) = 1.35.

**Table 2:** Relative risk of type 2 diabetes by degree and number of relatives with type 2 diabetes.

	Model A		Model B		Model C		Model D	
Variables	OR	95% CI (lower, upper)	OR	95% CI (lower, upper)	OR	95% CI (lower, upper)	OR	95% CI (lower, upper)
Number eligible FDRs^a^	0.90	0.89, 0.91	0.90	0.89, 0.91	0.91	0.90, 0.92	0.91	0.90, 0.92
Number eligible FDRs with type 2 diabetes								
1 vs. 0	1.98	1.91, 2.06	1.98	1.91, 2.06	1.76	1.70, 1.83	1.76	1.70, 1.83
2+ vs. 0	4.24	4.04, 4.44	4.24	4.04, 4.45	3.31	3.15, 3.48	3.31	3.16, 3.48
Number eligible SDRs^b^	0.99	0.99, 1.00	0.99	0.99, 1.00	0.99	0.99, 1.00	0.99	0.99, 1.00
Number eligible SDRs with type 2 diabetes								
1 vs. 0	1.10	1.04, 1.16	1.10	1.04, 1.16	1.10	1.04, 1.16	1.10	1.04, 1.16
2+ vs. 0	1.36	1.29, 1.43	1.36	1.29, 1.43	1.32	1.25, 1.39	1.32	1.25, 1.39
Number eligible FCs^c^	0.99	0.99, 0.99	0.99	0.99, 0.99	0.99	0.99, 1.00	0.99	0.99, 1.00
Number eligible FCs with type 2 diabetes								
1 vs. 0	1.10	1.05, 1.15	1.10	1.05, 1.15	1.07	1.02, 1.12	1.07	1.02, 1.12
2+ vs. 0	1.35	1.28, 1.42	1.35	1.27, 1.42	1.28	1.21, 1.35	1.28	1.21, 1.35
Male vs. female	0.71	0.69, 0.74	0.71	0.69, 0.74	0.66	0.64, 0.68	0.66	0.64, 0.69
Birth year	0.93	0.93, 0.94	0.93	0.93, 0.94	0.93	0.92, 0.93	0.93	0.92, 0.93
Whether White								
No vs. yes	1.56	1.47, 1.67	1.56	1.46, 1.66	1.37	1.28, 1.46	1.37	1.28, 1.46
Unknown vs. yes	0.67	0.26, 1.39	0.67	0.26, 1.38	0.78	0.31, 1.61	0.78	0.30, 1.61
Hispanic status								
Yes vs no	1.29	1.23, 1.36	1.29	1.23, 1.36	1.23	1.17, 1.30	1.23	1.17, 1.30
Unknown vs yes	0.28	0.25, 0.31	0.28	0.25, 0.31	0.29	0.26, 0.32	0.29	0.26, 0.32
Parental education								
No HS vs. HS	1.23	1.13, 1.34	1.23	1.13, 1.34	1.18	1.08, 1.28	1.18	1.08, 1.28
Some college vs. HS	0.88	0.85, 0.92	0.88	0.85, 0.92	0.92	0.88, 0.96	0.92	0.88, 0.96
College grad vs. HS	0.77	0.72, 0.81	0.77	0.72, 0.81	0.84	0.80, 0.89	0.84	0.79, 0.89
Postcollege vs. HS	0.75	0.71, 0.79	0.75	0.71, 0.79	0.84	0.80, 0.89	0.84	0.80, 0.89
Unknown vs. HS	0.79	0.61, 1.01	0.79	0.61, 1.01	0.83	0.64, 1.06	0.83	0.64, 1.06
Maximum CCI before age 30								
1 vs. 0			1.06	0.93, 1.21			1.05	0.91, 1.19
2 vs. 0			1.13	0.88, 1.43			1.14	0.89, 1.45
3–4 vs. 0			0.84	0.66, 1.06			0.84	0.66, 1.06
5+ vs. 0			0.33	0.12, 0.73			0.32	0.11, 0.69
Ever obese before age 30								
No vs Yes					0.24	0.23, 0.25	0.24	0.23, 0.25
Unknown vs Yes					0.46	0.44, 0.49	0.46	0.44, 0.49

Abbreviations: BMI, body mass index; CCI, Charlson Comorbidity Index; CI, confidence interval; FC, first cousin; FDR, first‐degree relative; HS, high school; OR, odds ratio; SDR, second‐degree relative.

^a^ FDRs, which include parents and siblings.

^b^ SDRs, which include aunts, uncles, grandparents, nieces, nephews, and half siblings.

^c^ FCs, which represent a portion of third‐degree relatives.

### 3.3 Logistic regressions: Mediating effects of health status and obesity

For Model B, the potentially mediating effects of health status as measured by the CCI are considered. The risk estimates associated with a family history remain largely unchanged from those in Model A that excluded CCI. Persons at the two highest comorbidity scores, which represent very poor health, show inconsistent effects: CCI scores of 3–4 are insignificant and scores of 5+ are less likely to be diagnosed with type 2 diabetes, likely a reflection of very small sample sizes.

**Figure 1:**
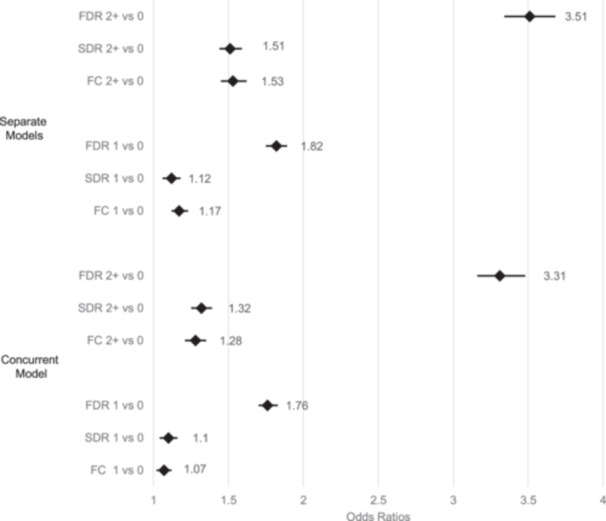
Forest plot of odds ratios of type 2 diabetes associated with the number and type of affected relatives. Concurrent model means the effects of first‐degree relative (FDR), which include parents and siblings, second‐degree relative (SDR), and first cousin (FC) are included in the same model. Separate models mean the effects of FDR, SDR, and FC are included alone in the distinct models. 1 versus 0 affected relative refers to a contrast between having a single relative with type 2 Diabetes Mellitus (T2DM) versus none. 2+ versus 0 affected relatives refer to a contrast between having two more of relatives with T2DM versus none. Risk estimates are based on Model D in Tables 2 and Supporting Information S1: Tables S1–S3.

For Model C, CCI is removed, and an indicator of whether an individual is obese before age 30 is introduced. Model C provides evidence of stronger mediating effects of obesity. The most noteworthy influence is on the association with FDRs where its risk for 2+ FDRs is attenuated from RR = 4.24 (Model A) to RR = 3.31 (Model C). This result suggests that the individual's own obesity is associated with their family history and partly explains the association between this history and the individual's own risk of type 2 diabetes. Model D, where both comorbidity and obesity are included concurrently, yields risk estimates nearly identical to Model C, again reflecting primarily the mediating influence of obesity. Model D shows that individuals with 2+ FDRs with type 2 diabetes have a significant risk of type 2 diabetes in relation to those with none (RR = 3.31, 95% CI: 3.16, 3.48). Those with 2+ affected versus no affected SDRs or FCs had significant but smaller risks: RR (SDR) = 1.32 (95% CI: 1.25, 1.39) and RR (FC) = 1.28 (95% CI: 1.21, 1.35). For our primary findings, the RRs are summarized in a Forest Plot (Figure 1).

### 3.4 Logistic regressions: main effects of family history estimated separately

In Supporting Information S1: Tables S1–S3, comparable risk estimates are reported where the effects of family history are estimated separately for FDRs, SDRs, and FCs, respectively (see also Figure 1). In general, each of these family history variables remain significant and parallel the results shown in Table 2: closer kin are associated with larger RRs than those more distantly related, larger numbers of affected relatives are associated with greater risks, as well as the risks of SDRs and FCs being similar. The attenuation of the RRs is seen for all of the family history variables (FDR, SDR, FC) when they are included concurrently but is most dramatic, in percentage terms, for the SDRs and FC (Figure 1). Using Model D as the basis for this comparison, the RR for 2+ affected SDRs in the smaller (separate) model is 1.51 (Supporting Information S1: Table S2), which then drops to 1.32 in the larger, concurrent model (Table 2). Similarly, for FCs, the RR changes from 1.53 for 2+ affected relatives (Supporting Information S1: Table S3) to 1.28 (Table 2). For FDRs, the comparable change in RR goes from 3.51 (Supporting Information S1: Table S1) to 3.31 (Table 2).

### 3.5 Logistic regressions: Stratified analyses by sex and Hispanic status

**Figure 2:**
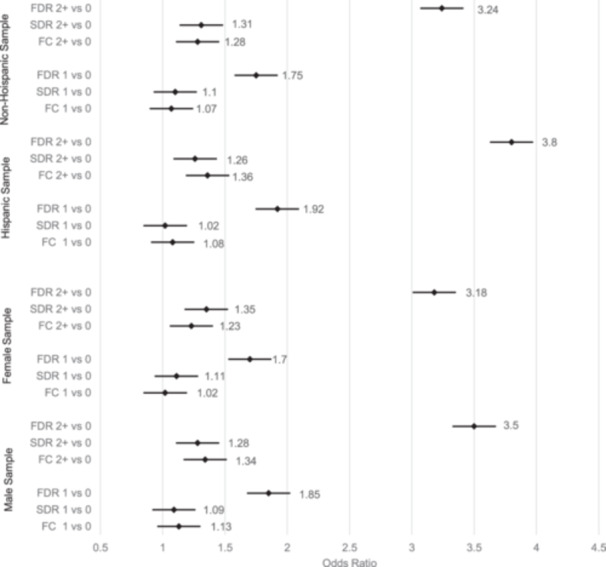
Forest plot of odds ratios of type 2 diabetes associated with the number and type of affected relatives in the sex‐ and Hispanic ethnicity‐specific subsamples. 1 versus 0 affected relative refers to a contrast between having a single relative with type 2 diabetes mellitus (T2DM) versus none. 2+ versus 0 affected relatives refer to a contrast between having two more of relatives with T2DM versus none. Risk estimates are based on Model D in Supporting Information S1: Tables S4, S5, S7, and S8. FC, first cousin; FDR, first‐degree relative, which include parents and siblings; SDR, second‐degree relative, which include aunts, uncles, grandparents, nieces, nephews, and half siblings.

Sex‐specific results are summarized in Figure 2. The qualitative patterns observed for the full sample are found in large measure in the male and female subsamples (e.g., risks are greatest with 2+ affected FDRs, Model D). In sex‐specific models, larger RRs are experienced for males (2+ FDRs RR = 3.55) (Supporting Information S1: Table S4) than for females (2+ FDRs RR = 3.18) (Supporting Information S1: Table S5). In the pooled all‐sex sample, evidence of effect modification is found where the risks associated with more affected FDRs and FCs are greater for males than females (*p* ≤ 0.05) (Supporting Information S1: Table S6).

There is only suggestive evidence that the risks associated with family history is stronger for Hispanic than non‐Hispanic individuals (Figure 2). Hispanic individuals with 2+ affected FDRs are nearly four times more likely to be diagnosed with type 2 diabetes than those with no affected FDRs (RR = 3.80) (Model D, Supporting Information S1: Table S7). For the non‐Hispanic sample, the comparable risk estimate is RR = 3.24 (Supporting Information S1: Table S8). In the pooled Hispanic and non‐Hispanic sample, the interaction term between having 2+ affected FDR and Hispanic status has a *p* value of 0.08 with Hispanic status amplifying the risk (Supporting Information S1: Table S9).

## 4 DISCUSSION

This study examines the role of family history on the risk of type 2 diabetes for a statewide sample of young and middle‐aged adults. Strong evidence is presented that shows an excess risk of type 2 diabetes associated with having multiple FDRs. FDRs with type 2 diabetes were shown to be associated with larger risks compared to the association of affected SDRs and FCs. This is not surprising given that FDRs share both 50% of their genes as well as their familial environments during the individual's childhood. Above and beyond the influence of FDRs, there is an added risk, albeit at a lower level, of having multiple affected SDRs and FCs. These consistent and significant associations are observed after controlling for important confounders including birth year, sex, race, Hispanic ethnicity, and parental education.

While type 2 diabetes rates are higher for females than males in our sample, the association between family history and the risk of type 2 diabetes is greater for males than that for females, possible due to genetic mechanisms.^22^, ^32^ One possible behavioral explanation for this pattern is that while males generally seek healthcare at lower rates than females, a possible factor that may encourage males to increase healthcare utilization is a stronger diabetes family history. This may lead to more type 2 diabetes diagnoses for men than they would have received otherwise.^33^, ^34^ This same mechanism may not be as potent for females since they are generally using the healthcare system more already. The suggestive evidence of a greater risk attributable to family history among Hispanic individuals requires more research with the inclusion of a more diverse set of national‐origin Hispanic individuals.

Health status and obesity are examined with respect to their potential mediating effects on the association between a family history and the risk of type 2 diabetes. Obesity is shown to have a mediating effect on the association, indicating that a possible mechanism linking family history to one's own risk of type 2 diabetes is via the individual's own obesity history.^35^ This result suggests that a family history of type 2 diabetes may influence the risk of obesity for young and middle‐aged adults, and once obesity is controlled for, the effects of that family history diminished. It is also likely that there is a constellation of parental or familial obesity that occurs along with parental or familial diabetes. An individual's own obesity may therefore be associated with not only their familial history of diabetes but it may also reflect a family history of excess weight. Future work will benefit from examining both BMI and diabetes in families to further our understanding of the possible dual role of both of these phenotypes on the risk of type 2 diabetes in young and middle‐aged adults.

The influence of SDRs was expected to have a stronger association with the risk of type 2 diabetes than the influence of FCs. Instead, the risks due to affected SDRs and FCs were very similar. For SDRs, comprising uncles, aunts, nephews, nieces, grandparents, and half siblings, many come from an older generation (aunts/uncles, grandparents) where diabetes and obesity rates were lower and where they share only 25% of their genes. For the nieces and nephews, they are younger and contribute less to the count of *affected* SDRs though type 2 diabetes at these young ages may signify stronger genetic risk. FCs are numerous and are roughly the same generation as the subjects and share only 12.5% of their genes. They do share some common childhood environments that are enhanced by having grown up in the same larger social environment and historical era that is associated with higher age‐specific rates of diabetes and obesity. But by being younger, affected FCs (a relative rarity) may also represent a stronger familial genetic risk.

The study has some limitations worth noting. First, the analysis focuses on individuals who are living in the urban core of Utah. How a different urban setting or rural context may influence the pattern of familial risk is not addressable here. It is noteworthy that the focal individuals are from urban areas, but the relatives themselves lived in all geographic settings in the state. Second, some research has shown that earlier age at diagnosis of type 2 diabetes for FDRs was associated with a greater risk of type 2 diabetes in relation to such relatives who were diagnosed at older ages.^36^ Given the nature of the UPDB, we were unable to identify the exact age of diagnosis of all the relatives. Third, while the UPDB provides deep genealogical and medical information, we were able to ascertain type 2 diabetes diagnoses on the vast majority of relatives but not all. Fourth, we did not consider the role that a spousal history of type 2 diabetes has and how it may illustrate the distinct roles of shared marital environments versus genetic effects on the risk of type 2 diabetes.^37^, ^38^ Finally, given the nature of the UPDB, the analysis plan could not directly account for the possible influence of lifestyle and diet as these measures were not available. At the same time, attention was given to the role of obesity which indirectly captures some of the variation attributable to physical activity and dietary intake.

Our study also offers several advantages and strengths. First, the risk estimates are based on a population database that includes all persons in the urban core of Utah who meet the age restrictions. This vastly reduces nonresponse and sampling error and exploits the objective diagnostic information derived from electronic medical records. Second, the analysis focuses on type 2 diabetes risk which has been an understudied but growing problem among younger and middle‐aged adults. Third, the UPDB permits an analysis of intergenerational risk given its extensive genealogical structure that connects relatives to each other. Fourth, we are able to assess the role of additional medical and demographic features that serve to sharpen our risks estimates. These additional variables include indicators of BMI, a comprehensive health status index, parental education, and ethnicity and racial measures.

Our findings regarding the relationship between affected FDRs and risk of type 2 diabetes are consistent with prior studies that link diabetes risk to genetics. Yet, our findings also suggest environmental and/or health‐seeking factors may play important roles in some of the differences we observe. For instance, our findings about the (unexpected) similar effects of affected SDRs and FCs point to the need for further research to assess if family history of type 2 diabetes confers differential risk by age and birth cohorts. Similarly, future research should examine the manner in whether gender differences in type 2 diabetes risk attributable to FDRs is genetic or a result of behavioral differences. Research in both of these areas could help target preventive efforts going forward.

## AUTHOR CONTRIBUTIONS

All authors contributed to the study's conception and design. Obtaining human subjects approval for access to the data was done by Ken R. Smith and Lori Kowaleski‐Jones. Construction of the data files was done by Huong Meeks. Model development and statistical analysis was done by Huong Meeks and Ken R. Smith. All authors contributed to the manuscript preparation and editing.

## CONFLICT OF INTEREST STATEMENT

The authors declare no conflict of interest.

## ETHICS STATEMENT

This study was approved by the University of Utah's Institutional Review Board (IRB # IRB_00108522) and the Resource for Genetic and Epidemiologic Research, the body that oversees access to the Utah Population Database.

## Supporting information

Supporting information.

## Data Availability

The Utah Resource for Genetic and Epidemiologic Research (RGE), established in 1982 by Executive Order of the Governor of Utah, administers access to the Utah Population Database (UPDB), the data used here, through a review process of all proposals using UPDB data. The protection of privacy and confidentiality of individuals represented in these records has been negotiated with agreements between RGE and data contributors. Data from the UPDB are available only for approved health‐related research studies, and access is project specific and granted after review and approval by an RGE oversight committee and the University of Utah's IRB. This process allows researchers with approved protocols to use the data, a process that has proven effective and successful as evidenced by hundreds of approved studies that have relied on the UPDB. More information about access to UPDB is located here https://uofuhealth.utah.edu/huntsman/utah-population-database/data/access.
