# Supplementary material for: Family history of type 2 diabetes and the risk of type 2 diabetes among young and middle‐aged adults
Source: Chronic Dis Transl Med. 2024 Jul 23;11(1):46–56. doi: 10.1002/cdt3.147 (PMC11880113; doi:10.1002/cdt3.147)
Supplement: Supplementary file 1 — Supporting information. [file CDT3-11-46-s001.docx]

Table S1: Relative risk of family history of type 2 diabetes by degree of relatives and number of relatives with type 2 diabetes; Effects of First-Degree Relatives (FDR) Only.

|  | **Model A** | | | **Model B** | | | **Model C** | | | **Model D** | | |
| --- | --- | --- | --- | --- | --- | --- | --- | --- | --- | --- | --- | --- |
| **Characteristic** | **OR**^1^ | **95% CI**^1^ | **p-value** | **OR**^1^ | **95% CI**^1^ | **p-value** | **OR**^1^ | **95% CI**^1^ | **p-value** | **OR**^1^ | **95% CI**^1^ | **p-value** |
| Sex | 0.71 | 0.69, 0.74 | **<0.001** | 0.71 | 0.69, 0.74 | **<0.001** | 0.66 | 0.64, 0.68 | **<0.001** | 0.66 | 0.64, 0.68 | **<0.001** |
| Birth year | 0.93 | 0.93, 0.93 | **<0.001** | 0.93 | 0.93, 0.93 | **<0.001** | 0.93 | 0.92, 0.93 | **<0.001** | 0.93 | 0.92, 0.93 | **<0.001** |
| Whether Caucasian |  |  |  |  |  |  |  |  |  |  |  |  |
| Yes | — | — |  | — | — |  | — | — |  | — | — |  |
| No | 1.54 | 1.45, 1.64 | **<0.001** | 1.54 | 1.45, 1.64 | **<0.001** | 1.35 | 1.26, 1.44 | **<0.001** | 1.35 | 1.26, 1.44 | **<0.001** |
| Unknown | 0.64 | 0.25, 1.33 | 0.287 | 0.64 | 0.25, 1.32 | 0.285 | 0.75 | 0.29, 1.55 | 0.488 | 0.75 | 0.29, 1.55 | 0.486 |
| Whether Hispanic |  |  |  |  |  |  |  |  |  |  |  |  |
| No | — | — |  | — | — |  | — | — |  | — | — |  |
| Yes | 1.30 | 1.24, 1.37 | **<0.001** | 1.30 | 1.24, 1.37 | **<0.001** | 1.24 | 1.17, 1.30 | **<0.001** | 1.24 | 1.17, 1.30 | **<0.001** |
| Unknown | 0.27 | 0.25, 0.30 | **<0.001** | 0.27 | 0.25, 0.30 | **<0.001** | 0.29 | 0.26, 0.32 | **<0.001** | 0.29 | 0.26, 0.32 | **<0.001** |
| Maximum parental education level |  |  |  |  |  |  |  |  |  |  |  |  |
| HS degree | — | — |  | — | — |  | — | — |  | — | — |  |
| Less than HS | 1.22 | 1.12, 1.33 | **<0.001** | 1.22 | 1.12, 1.33 | **<0.001** | 1.17 | 1.07, 1.27 | **<0.001** | 1.17 | 1.07, 1.27 | **<0.001** |
| Some college | 0.87 | 0.84, 0.91 | **<0.001** | 0.87 | 0.84, 0.91 | **<0.001** | 0.91 | 0.87, 0.95 | **<0.001** | 0.91 | 0.87, 0.95 | **<0.001** |
| College degree | 0.74 | 0.71, 0.79 | **<0.001** | 0.74 | 0.70, 0.79 | **<0.001** | 0.82 | 0.78, 0.87 | **<0.001** | 0.82 | 0.78, 0.87 | **<0.001** |
| Post college | 0.73 | 0.69, 0.77 | **<0.001** | 0.73 | 0.69, 0.77 | **<0.001** | 0.82 | 0.78, 0.86 | **<0.001** | 0.82 | 0.78, 0.86 | **<0.001** |
| Unknown | 0.73 | 0.56, 0.93 | **0.013** | 0.73 | 0.57, 0.93 | **0.013** | 0.77 | 0.60, 0.99 | **0.044** | 0.77 | 0.60, 0.98 | **0.044** |
| Number of eligible FDRs | 0.89 | 0.88, 0.90 | **<0.001** | 0.89 | 0.88, 0.90 | **<0.001** | 0.91 | 0.90, 0.92 | **<0.001** | 0.91 | 0.90, 0.92 | **<0.001** |
| Number of eligible FDRs with T2DM |  |  |  |  |  |  |  |  |  |  |  |  |
| 0 | — | — |  | — | — |  | — | — |  | — | — |  |
| 1 | 2.05 | 1.98, 2.13 | **<0.001** | 2.05 | 1.98, 2.13 | **<0.001** | 1.82 | 1.75, 1.89 | **<0.001** | 1.82 | 1.75, 1.89 | **<0.001** |
| 2+ | 4.54 | 4.33, 4.76 | **<0.001** | 4.55 | 4.34, 4.76 | **<0.001** | 3.51 | 3.34, 3.68 | **<0.001** | 3.51 | 3.34, 3.68 | **<0.001** |
| Maximum CCI excluding diabetes before age 30 |  |  |  |  |  |  |  |  |  |  |  |  |
| 0 |  |  |  | — | — |  |  |  |  | — | — |  |
| 1 |  |  |  | 1.06 | 0.93, 1.21 | 0.364 |  |  |  | 1.05 | 0.92, 1.19 | 0.484 |
| 2 |  |  |  | 1.15 | 0.89, 1.45 | 0.270 |  |  |  | 1.16 | 0.90, 1.47 | 0.244 |
| 3-4 |  |  |  | 0.84 | 0.66, 1.06 | 0.154 |  |  |  | 0.84 | 0.65, 1.05 | 0.142 |
| 5+ |  |  |  | 0.33 | 0.12, 0.72 | **0.014** |  |  |  | 0.31 | 0.11, 0.68 | **0.010** |
| Ever obese before age 30 (BMI) |  |  |  |  |  |  |  |  |  |  |  |  |
| Yes |  |  |  |  |  |  | — | — |  | — | — |  |
| No |  |  |  |  |  |  | 0.23 | 0.22, 0.24 | **<0.001** | 0.23 | 0.22, 0.24 | **<0.001** |
| Unknown |  |  |  |  |  |  | 0.46 | 0.43, 0.49 | **<0.001** | 0.46 | 0.43, 0.49 | **<0.001** |
| ^1^OR = Odds Ratio, CI = Confidence Interval  Model A controls for number of eligible relatives, sex, birth year, non-white race, Hispanic ethnicity, highest parental education.  Model B additionally controls for co-morbidity  Model C excludes comorbidity and adds obesity;  Model D adds co-morbidity and obesity controls. | | | | | | | | | | | | |

Table S2: Relative risk of family history of type 2 diabetes by degree of relatives and number of relatives with type 2 diabetes; Effects of Second-Degree Relatives (SDR) Only.

|  | **Model A** | | | **Model B** | | | **Model C** | | | **Model D** | | |
| --- | --- | --- | --- | --- | --- | --- | --- | --- | --- | --- | --- | --- |
| **Characteristic** | **OR**^1^ | **95% CI**^1^ | **p-value** | **OR**^1^ | **95% CI**^1^ | **p-value** | **OR**^1^ | **95% CI**^1^ | **p-value** | **OR**^1^ | **95% CI**^1^ | **p-value** |
| Sex | 0.72 | 0.69, 0.74 | **<0.001** | 0.72 | 0.69, 0.74 | **<0.001** | 0.66 | 0.63, 0.68 | **<0.001** | 0.66 | 0.63, 0.68 | **<0.001** |
| Birth year | 0.92 | 0.92, 0.92 | **<0.001** | 0.92 | 0.92, 0.92 | **<0.001** | 0.92 | 0.91, 0.92 | **<0.001** | 0.92 | 0.92, 0.92 | **<0.001** |
| Whether Caucasian |  |  |  |  |  |  |  |  |  |  |  |  |
| Yes | — | — |  | — | — |  | — | — |  | — | — |  |
| No | 1.85 | 1.73, 1.97 | **<0.001** | 1.85 | 1.73, 1.97 | **<0.001** | 1.55 | 1.45, 1.65 | **<0.001** | 1.55 | 1.45, 1.65 | **<0.001** |
| Unknown | 0.69 | 0.27, 1.42 | 0.368 | 0.69 | 0.27, 1.42 | 0.366 | 0.81 | 0.32, 1.67 | 0.603 | 0.80 | 0.32, 1.67 | 0.601 |
| Whether Hispanic |  |  |  |  |  |  |  |  |  |  |  |  |
| No | — | — |  | — | — |  | — | — |  | — | — |  |
| Yes | 1.45 | 1.38, 1.52 | **<0.001** | 1.45 | 1.38, 1.52 | **<0.001** | 1.34 | 1.28, 1.41 | **<0.001** | 1.34 | 1.28, 1.41 | **<0.001** |
| Unknown | 0.27 | 0.24, 0.30 | **<0.001** | 0.27 | 0.24, 0.30 | **<0.001** | 0.28 | 0.26, 0.32 | **<0.001** | 0.28 | 0.26, 0.32 | **<0.001** |
| Maximum parental education level |  |  |  |  |  |  |  |  |  |  |  |  |
| HS degree | — | — |  | — | — |  | — | — |  | — | — |  |
| Less than HS | 1.30 | 1.19, 1.41 | **<0.001** | 1.30 | 1.19, 1.41 | **<0.001** | 1.22 | 1.12, 1.32 | **<0.001** | 1.22 | 1.12, 1.32 | **<0.001** |
| Some college | 0.85 | 0.82, 0.89 | **<0.001** | 0.85 | 0.82, 0.89 | **<0.001** | 0.90 | 0.86, 0.94 | **<0.001** | 0.90 | 0.86, 0.94 | **<0.001** |
| College degree | 0.68 | 0.64, 0.72 | **<0.001** | 0.68 | 0.64, 0.72 | **<0.001** | 0.78 | 0.74, 0.82 | **<0.001** | 0.78 | 0.74, 0.82 | **<0.001** |
| Post college | 0.65 | 0.62, 0.68 | **<0.001** | 0.65 | 0.62, 0.68 | **<0.001** | 0.76 | 0.72, 0.80 | **<0.001** | 0.76 | 0.72, 0.80 | **<0.001** |
| Unknown | 0.82 | 0.64, 1.04 | 0.118 | 0.82 | 0.64, 1.04 | 0.119 | 0.85 | 0.66, 1.08 | 0.206 | 0.85 | 0.66, 1.08 | 0.207 |
| Number of eligible SDRs | 0.98 | 0.97, 0.98 | **<0.001** | 0.98 | 0.97, 0.98 | **<0.001** | 0.98 | 0.98, 0.99 | **<0.001** | 0.98 | 0.98, 0.99 | **<0.001** |
| Number of eligible SDRs with T2DM |  |  |  |  |  |  |  |  |  |  |  |  |
| 0 | — | — |  | — | — |  | — | — |  | — | — |  |
| 1 | 1.13 | 1.07, 1.19 | **<0.001** | 1.13 | 1.07, 1.19 | **<0.001** | 1.12 | 1.06, 1.18 | **<0.001** | 1.12 | 1.06, 1.18 | **<0.001** |
| 2+ | 1.61 | 1.53, 1.68 | **<0.001** | 1.61 | 1.53, 1.68 | **<0.001** | 1.51 | 1.44, 1.59 | **<0.001** | 1.51 | 1.44, 1.59 | **<0.001** |
| Maximum CCI excluding diabetes before age 30 |  |  |  |  |  |  |  |  |  |  |  |  |
| 0 |  |  |  | — | — |  |  |  |  | — | — |  |
| 1 |  |  |  | 1.03 | 0.91, 1.18 | 0.608 |  |  |  | 1.02 | 0.89, 1.16 | 0.763 |
| 2 |  |  |  | 1.13 | 0.88, 1.43 | 0.303 |  |  |  | 1.14 | 0.89, 1.45 | 0.281 |
| 3-4 |  |  |  | 0.86 | 0.67, 1.07 | 0.193 |  |  |  | 0.85 | 0.67, 1.07 | 0.189 |
| 5+ |  |  |  | 0.34 | 0.12, 0.74 | **0.017** |  |  |  | 0.33 | 0.12, 0.72 | **0.014** |
| Ever obese before age 30 (BMI) |  |  |  |  |  |  |  |  |  |  |  |  |
| Yes |  |  |  |  |  |  | — | — |  | — | — |  |
| No |  |  |  |  |  |  | 0.20 | 0.19, 0.21 | **<0.001** | 0.20 | 0.19, 0.21 | **<0.001** |
| Unknown |  |  |  |  |  |  | 0.42 | 0.39, 0.44 | **<0.001** | 0.42 | 0.39, 0.44 | **<0.001** |
| ^1^OR = Odds Ratio, CI = Confidence Interval  Model A controls for number of eligible relatives, sex, birth year, non-white race, Hispanic ethnicity, highest parental education.  Model B additionally controls for co-morbidity  Model C excludes comorbidity and adds obesity;  Model D adds co-morbidity and obesity controls. | | | | | | | | | | | | |

Table S3: Relative risk of family history of type 2 diabetes by degree of relatives and number of relatives with type 2 diabetes; Effects of First Cousins (FC) Only.

|  | **Model A** | | | **Model B** | | | **Model C** | | | **Model D** | | |
| --- | --- | --- | --- | --- | --- | --- | --- | --- | --- | --- | --- | --- |
| **Characteristic** | **OR**^1^ | **95% CI**^1^ | **p-value** | **OR**^1^ | **95% CI**^1^ | **p-value** | **OR**^1^ | **95% CI**^1^ | **p-value** | **OR**^1^ | **95% CI**^1^ | **p-value** |
| Sex | 0.72 | 0.70, 0.74 | **<0.001** | 0.72 | 0.70, 0.74 | **<0.001** | 0.66 | 0.64, 0.68 | **<0.001** | 0.66 | 0.64, 0.68 | **<0.001** |
| Birth year | 0.92 | 0.92, 0.93 | **<0.001** | 0.92 | 0.92, 0.93 | **<0.001** | 0.92 | 0.92, 0.92 | **<0.001** | 0.92 | 0.92, 0.92 | **<0.001** |
| Whether Caucasian |  |  |  |  |  |  |  |  |  |  |  |  |
| Yes | — | — |  | — | — |  | — | — |  | — | — |  |
| No | 1.74 | 1.64, 1.86 | **<0.001** | 1.74 | 1.64, 1.86 | **<0.001** | 1.47 | 1.38, 1.57 | **<0.001** | 1.47 | 1.38, 1.57 | **<0.001** |
| Unknown | 0.63 | 0.25, 1.31 | 0.273 | 0.63 | 0.25, 1.31 | 0.271 | 0.76 | 0.30, 1.56 | 0.501 | 0.75 | 0.30, 1.56 | 0.498 |
| Whether Hispanic |  |  |  |  |  |  |  |  |  |  |  |  |
| No | — | — |  | — | — |  | — | — |  | — | — |  |
| Yes | 1.40 | 1.33, 1.47 | **<0.001** | 1.40 | 1.33, 1.47 | **<0.001** | 1.31 | 1.24, 1.38 | **<0.001** | 1.31 | 1.24, 1.38 | **<0.001** |
| Unknown | 0.27 | 0.24, 0.30 | **<0.001** | 0.27 | 0.24, 0.30 | **<0.001** | 0.28 | 0.26, 0.31 | **<0.001** | 0.28 | 0.26, 0.31 | **<0.001** |
| Maximum parental education level |  |  |  |  |  |  |  |  |  |  |  |  |
| HS degree | — | — |  | — | — |  | — | — |  | — | — |  |
| Less than HS | 1.25 | 1.15, 1.35 | **<0.001** | 1.25 | 1.15, 1.35 | **<0.001** | 1.18 | 1.09, 1.28 | **<0.001** | 1.18 | 1.08, 1.28 | **<0.001** |
| Some college | 0.87 | 0.84, 0.91 | **<0.001** | 0.87 | 0.84, 0.91 | **<0.001** | 0.92 | 0.88, 0.96 | **<0.001** | 0.91 | 0.88, 0.96 | **<0.001** |
| College degree | 0.70 | 0.66, 0.74 | **<0.001** | 0.70 | 0.66, 0.74 | **<0.001** | 0.80 | 0.75, 0.84 | **<0.001** | 0.80 | 0.75, 0.84 | **<0.001** |
| Post college | 0.67 | 0.63, 0.70 | **<0.001** | 0.67 | 0.63, 0.70 | **<0.001** | 0.77 | 0.73, 0.81 | **<0.001** | 0.77 | 0.73, 0.81 | **<0.001** |
| Unknown | 0.75 | 0.58, 0.96 | **0.025** | 0.75 | 0.58, 0.96 | **0.025** | 0.79 | 0.61, 1.01 | 0.068 | 0.79 | 0.61, 1.01 | 0.068 |
| Number of eligible FCs | 0.99 | 0.99, 0.99 | **<0.001** | 0.99 | 0.99, 0.99 | **<0.001** | 0.99 | 0.99, 0.99 | **<0.001** | 0.99 | 0.99, 0.99 | **<0.001** |
| Number of eligible FCs with T2DM |  |  |  |  |  |  |  |  |  |  |  |  |
| 0 | — | — |  | — | — |  | — | — |  | — | — |  |
| 1 | 1.22 | 1.17, 1.28 | **<0.001** | 1.22 | 1.17, 1.28 | **<0.001** | 1.17 | 1.12, 1.23 | **<0.001** | 1.17 | 1.12, 1.23 | **<0.001** |
| 2+ | 1.69 | 1.60, 1.78 | **<0.001** | 1.69 | 1.60, 1.78 | **<0.001** | 1.53 | 1.45, 1.62 | **<0.001** | 1.53 | 1.45, 1.62 | **<0.001** |
| Maximum CCI excluding diabetes before age 30 |  |  |  |  |  |  |  |  |  |  |  |  |
| 0 |  |  |  | — | — |  |  |  |  | — | — |  |
| 1 |  |  |  | 1.04 | 0.91, 1.18 | 0.558 |  |  |  | 1.03 | 0.90, 1.17 | 0.706 |
| 2 |  |  |  | 1.13 | 0.88, 1.43 | 0.311 |  |  |  | 1.14 | 0.88, 1.44 | 0.297 |
| 3-4 |  |  |  | 0.86 | 0.67, 1.08 | 0.202 |  |  |  | 0.86 | 0.67, 1.08 | 0.209 |
| 5+ |  |  |  | 0.34 | 0.12, 0.74 | **0.017** |  |  |  | 0.33 | 0.12, 0.71 | **0.013** |
| Ever obese before age 30 (BMI) |  |  |  |  |  |  |  |  |  |  |  |  |
| Yes |  |  |  |  |  |  | — | — |  | — | — |  |
| No |  |  |  |  |  |  | 0.20 | 0.19, 0.21 | **<0.001** | 0.20 | 0.19, 0.21 | **<0.001** |
| Unknown |  |  |  |  |  |  | 0.41 | 0.39, 0.44 | **<0.001** | 0.41 | 0.39, 0.44 | **<0.001** |
| ^1^OR = Odds Ratio, CI = Confidence Interval  Model A controls for number of eligible relatives, sex, birth year, non-white race, Hispanic ethnicity, highest parental education.  Model B additionally controls for co-morbidity  Model C excludes comorbidity and adds obesity;  Model D adds co-morbidity and obesity controls. | | | | | | | | | | | | |

Table S4: Relative risk of family history of type 2 diabetes by degree and number of relatives with type 2 diabetes: Male Subsample

|  | **Model A** | | | **Model B** | | | **Model C** | | | **Model D** | | |
| --- | --- | --- | --- | --- | --- | --- | --- | --- | --- | --- | --- | --- |
| **Characteristic** | **OR**^1^ | **95% CI**^1^ | **p-value** | **OR**^1^ | **95% CI**^1^ | **p-value** | **OR**^1^ | **95% CI**^1^ | **p-value** | **OR**^1^ | **95% CI**^1^ | **p-value** |
| Birth year | 0.92 | 0.92, 0.93 | **<0.001** | 0.92 | 0.92, 0.93 | **<0.001** | 0.92 | 0.92, 0.93 | **<0.001** | 0.92 | 0.92, 0.93 | **<0.001** |
| Whether Caucasian |  |  |  |  |  |  |  |  |  |  |  |  |
| Yes | — | — |  | — | — |  | — | — |  | — | — |  |
| No | 1.58 | 1.43, 1.74 | **<0.001** | 1.58 | 1.43, 1.74 | **<0.001** | 1.36 | 1.23, 1.50 | **<0.001** | 1.36 | 1.23, 1.50 | **<0.001** |
| Unknown | 0.77 | 0.23, 1.83 | 0.601 | 0.76 | 0.23, 1.83 | 0.599 | 0.88 | 0.27, 2.10 | 0.798 | 0.88 | 0.27, 2.10 | 0.796 |
| Whether Hispanic |  |  |  |  |  |  |  |  |  |  |  |  |
| No | — | — |  | — | — |  | — | — |  | — | — |  |
| Yes | 1.28 | 1.18, 1.39 | **<0.001** | 1.28 | 1.18, 1.39 | **<0.001** | 1.22 | 1.12, 1.32 | **<0.001** | 1.22 | 1.12, 1.32 | **<0.001** |
| Unknown | 0.30 | 0.26, 0.34 | **<0.001** | 0.30 | 0.26, 0.34 | **<0.001** | 0.31 | 0.27, 0.36 | **<0.001** | 0.31 | 0.27, 0.36 | **<0.001** |
| Maximum parental education level |  |  |  |  |  |  |  |  |  |  |  |  |
| HS degree | — | — |  | — | — |  | — | — |  | — | — |  |
| Less than HS | 1.20 | 1.06, 1.37 | **0.005** | 1.20 | 1.06, 1.37 | **0.005** | 1.17 | 1.02, 1.33 | **0.020** | 1.17 | 1.02, 1.33 | **0.020** |
| Some college | 0.90 | 0.84, 0.97 | **0.003** | 0.90 | 0.84, 0.97 | **0.003** | 0.93 | 0.86, 0.99 | **0.027** | 0.93 | 0.86, 0.99 | **0.027** |
| College degree | 0.82 | 0.76, 0.90 | **<0.001** | 0.82 | 0.76, 0.89 | **<0.001** | 0.87 | 0.80, 0.95 | **0.002** | 0.87 | 0.80, 0.95 | **0.002** |
| Post college | 0.84 | 0.78, 0.91 | **<0.001** | 0.84 | 0.78, 0.91 | **<0.001** | 0.92 | 0.84, 0.99 | **0.032** | 0.92 | 0.84, 0.99 | **0.031** |
| Unknown | 0.84 | 0.56, 1.21 | 0.368 | 0.84 | 0.56, 1.21 | 0.371 | 0.88 | 0.58, 1.27 | 0.505 | 0.88 | 0.58, 1.27 | 0.504 |
| Number of eligible FDRs | 0.91 | 0.89, 0.92 | **<0.001** | 0.91 | 0.89, 0.92 | **<0.001** | 0.92 | 0.91, 0.94 | **<0.001** | 0.92 | 0.91, 0.94 | **<0.001** |
| Number of eligible FDRs with T2DM |  |  |  |  |  |  |  |  |  |  |  |  |
| 0 | — | — |  | — | — |  | — | — |  | — | — |  |
| 1 | 2.05 | 1.93, 2.17 | **<0.001** | 2.05 | 1.93, 2.17 | **<0.001** | 1.85 | 1.75, 1.96 | **<0.001** | 1.85 | 1.75, 1.96 | **<0.001** |
| 2+ | 4.37 | 4.06, 4.69 | **<0.001** | 4.37 | 4.07, 4.70 | **<0.001** | 3.50 | 3.25, 3.77 | **<0.001** | 3.50 | 3.26, 3.77 | **<0.001** |
| Number of eligible SDRs | 0.99 | 0.98, 0.99 | **<0.001** | 0.99 | 0.98, 0.99 | **<0.001** | 0.99 | 0.98, 1.00 | **0.002** | 0.99 | 0.98, 1.00 | **0.002** |
| Number of eligible SDRs with T2DM |  |  |  |  |  |  |  |  |  |  |  |  |
| 0 | — | — |  | — | — |  | — | — |  | — | — |  |
| 1 | 1.10 | 1.01, 1.19 | **0.025** | 1.10 | 1.01, 1.19 | **0.026** | 1.09 | 1.01, 1.19 | **0.030** | 1.09 | 1.01, 1.19 | **0.030** |
| 2+ | 1.33 | 1.23, 1.44 | **<0.001** | 1.33 | 1.23, 1.43 | **<0.001** | 1.29 | 1.19, 1.39 | **<0.001** | 1.28 | 1.19, 1.39 | **<0.001** |
| Number of eligible FCs | 0.99 | 0.99, 1.00 | **0.001** | 0.99 | 0.99, 1.00 | **0.001** | 1.00 | 0.99, 1.00 | **0.019** | 1.00 | 0.99, 1.00 | **0.019** |
| Number of eligible FCs with T2DM |  |  |  |  |  |  |  |  |  |  |  |  |
| 0 | — | — |  | — | — |  | — | — |  | — | — |  |
| 1 | 1.15 | 1.07, 1.23 | **<0.001** | 1.15 | 1.07, 1.23 | **<0.001** | 1.13 | 1.06, 1.22 | **<0.001** | 1.13 | 1.05, 1.22 | **<0.001** |
| 2+ | 1.40 | 1.29, 1.52 | **<0.001** | 1.40 | 1.29, 1.52 | **<0.001** | 1.34 | 1.23, 1.46 | **<0.001** | 1.34 | 1.23, 1.45 | **<0.001** |
| Maximum CCI excluding diabetes before age 30 |  |  |  |  |  |  |  |  |  |  |  |  |
| 0 |  |  |  | — | — |  |  |  |  | — | — |  |
| 1 |  |  |  | 0.99 | 0.80, 1.21 | 0.928 |  |  |  | 1.0 | 0.80, 1.22 | 0.961 |
| 2 |  |  |  | 1.37 | 0.96, 1.91 | 0.071 |  |  |  | 1.36 | 0.95, 1.90 | 0.081 |
| 3-4 |  |  |  | 0.93 | 0.63, 1.31 | 0.684 |  |  |  | 0.90 | 0.61, 1.28 | 0.592 |
| 5+ |  |  |  | 0.47 | 0.11, 1.23 | 0.191 |  |  |  | 0.42 | 0.10, 1.13 | 0.144 |
| Ever obese before age 30 (BMI) |  |  |  |  |  |  |  |  |  |  |  |  |
| Yes |  |  |  |  |  |  | — | — |  | — | — |  |
| No |  |  |  |  |  |  | 0.26 | 0.24, 0.27 | **<0.001** | 0.26 | 0.24, 0.27 | **<0.001** |
| Unknown |  |  |  |  |  |  | 0.53 | 0.49, 0.58 | **<0.001** | 0.53 | 0.49, 0.58 | **<0.001** |
| ^1^OR = Odds Ratio, CI = Confidence Interval  Model A controls for number of eligible relatives, sex, birth year, non-white race, Hispanic ethnicity, highest parental education.  Model B additionally controls for co-morbidity  Model C excludes comorbidity and adds obesity;  Model D adds co-morbidity and obesity controls. | | | | | | | | | | | | |

Table S5: Relative risk of family history of type 2 diabetes by degree and number of relatives with type 2 diabetes: Female Subsample

|  | **Model A** | | | **Model B** | | | **Model C** | | | **Model D** | | |
| --- | --- | --- | --- | --- | --- | --- | --- | --- | --- | --- | --- | --- |
| **Characteristic** | **OR**^1^ | **95% CI**^1^ | **p-value** | **OR**^1^ | **95% CI**^1^ | **p-value** | **OR**^1^ | **95% CI**^1^ | **p-value** | **OR**^1^ | **95% CI**^1^ | **p-value** |
| Birth year | 0.94 | 0.93, 0.94 | **<0.001** | 0.94 | 0.93, 0.94 | **<0.001** | 0.93 | 0.93, 0.94 | **<0.001** | 0.93 | 0.93, 0.94 | **<0.001** |
| Whether Caucasian |  |  |  |  |  |  |  |  |  |  |  |  |
| Yes | — | — |  | — | — |  | — | — |  | — | — |  |
| No | 1.55 | 1.43, 1.69 | **<0.001** | 1.55 | 1.43, 1.69 | **<0.001** | 1.38 | 1.27, 1.51 | **<0.001** | 1.38 | 1.27, 1.51 | **<0.001** |
| Unknown | 0.56 | 0.09, 1.79 | 0.418 | 0.56 | 0.09, 1.79 | 0.417 | 0.65 | 0.11, 2.10 | 0.555 | 0.65 | 0.11, 2.10 | 0.554 |
| Whether Hispanic |  |  |  |  |  |  |  |  |  |  |  |  |
| No | — | — |  | — | — |  | — | — |  | — | — |  |
| Yes | 1.30 | 1.22, 1.39 | **<0.001** | 1.30 | 1.22, 1.39 | **<0.001** | 1.24 | 1.16, 1.33 | **<0.001** | 1.24 | 1.16, 1.33 | **<0.001** |
| Unknown | 0.26 | 0.22, 0.30 | **<0.001** | 0.26 | 0.22, 0.30 | **<0.001** | 0.26 | 0.22, 0.31 | **<0.001** | 0.26 | 0.22, 0.31 | **<0.001** |
| Maximum parental education level |  |  |  |  |  |  |  |  |  |  |  |  |
| HS degree | — | — |  | — | — |  | — | — |  | — | — |  |
| Less than HS | 1.26 | 1.12, 1.40 | **<0.001** | 1.25 | 1.12, 1.40 | **<0.001** | 1.19 | 1.06, 1.33 | **0.002** | 1.19 | 1.06, 1.33 | **0.003** |
| Some college | 0.87 | 0.82, 0.92 | **<0.001** | 0.87 | 0.82, 0.92 | **<0.001** | 0.91 | 0.86, 0.97 | **0.002** | 0.91 | 0.86, 0.97 | **0.002** |
| College degree | 0.73 | 0.67, 0.78 | **<0.001** | 0.73 | 0.67, 0.78 | **<0.001** | 0.82 | 0.76, 0.88 | **<0.001** | 0.82 | 0.76, 0.88 | **<0.001** |
| Post college | 0.69 | 0.64, 0.74 | **<0.001** | 0.69 | 0.64, 0.74 | **<0.001** | 0.79 | 0.74, 0.85 | **<0.001** | 0.79 | 0.73, 0.85 | **<0.001** |
| Unknown | 0.76 | 0.54, 1.04 | 0.099 | 0.76 | 0.54, 1.04 | 0.099 | 0.80 | 0.57, 1.10 | 0.188 | 0.80 | 0.57, 1.10 | 0.187 |
| Number of eligible FDRs | 0.89 | 0.87, 0.90 | **<0.001** | 0.89 | 0.87, 0.90 | **<0.001** | 0.91 | 0.89, 0.92 | **<0.001** | 0.91 | 0.89, 0.92 | **<0.001** |
| Number of eligible FDRs with T2DM |  |  |  |  |  |  |  |  |  |  |  |  |
| 0 | — | — |  | — | — |  | — | — |  | — | — |  |
| 1 | 1.93 | 1.84, 2.03 | **<0.001** | 1.93 | 1.84, 2.03 | **<0.001** | 1.70 | 1.62, 1.79 | **<0.001** | 1.70 | 1.62, 1.79 | **<0.001** |
| 2+ | 4.14 | 3.89, 4.42 | **<0.001** | 4.15 | 3.89, 4.42 | **<0.001** | 3.18 | 2.97, 3.39 | **<0.001** | 3.18 | 2.98, 3.39 | **<0.001** |
| Number of eligible SDRs | 1.00 | 0.99, 1.00 | 0.474 | 1.00 | 0.99, 1.00 | 0.473 | 1.00 | 0.99, 1.00 | 0.240 | 1.00 | 0.99, 1.00 | 0.241 |
| Number of eligible SDRs with T2DM |  |  |  |  |  |  |  |  |  |  |  |  |
| 0 | — | — |  | — | — |  | — | — |  | — | — |  |
| 1 | 1.10 | 1.03, 1.18 | **0.005** | 1.10 | 1.03, 1.18 | **0.005** | 1.11 | 1.03, 1.19 | **0.005** | 1.11 | 1.03, 1.19 | **0.005** |
| 2+ | 1.39 | 1.30, 1.48 | **<0.001** | 1.39 | 1.30, 1.48 | **<0.001** | 1.35 | 1.26, 1.44 | **<0.001** | 1.35 | 1.26, 1.44 | **<0.001** |
| Number of eligible FCs | 0.99 | 0.99, 0.99 | **<0.001** | 0.99 | 0.99, 0.99 | **<0.001** | 0.99 | 0.99, 1.00 | **<0.001** | 0.99 | 0.99, 1.00 | **<0.001** |
| Number of eligible FCs with T2DM |  |  |  |  |  |  |  |  |  |  |  |  |
| 0 | — | — |  | — | — |  | — | — |  | — | — |  |
| 1 | 1.06 | 1.00, 1.13 | 0.051 | 1.06 | 1.00, 1.13 | 0.051 | 1.02 | 0.96, 1.09 | 0.513 | 1.02 | 0.96, 1.09 | 0.516 |
| 2+ | 1.31 | 1.21, 1.41 | **<0.001** | 1.31 | 1.21, 1.41 | **<0.001** | 1.23 | 1.14, 1.32 | **<0.001** | 1.23 | 1.14, 1.32 | **<0.001** |
| Maximum CCI excluding diabetes before age 30 |  |  |  |  |  |  |  |  |  |  |  |  |
| 0 |  |  |  | — | — |  |  |  |  | — | — |  |
| 1 |  |  |  | 1.11 | 0.94, 1.31 | 0.215 |  |  |  | 1.08 | 0.91, 1.28 | 0.364 |
| 2 |  |  |  | 0.96 | 0.67, 1.33 | 0.826 |  |  |  | 0.99 | 0.69, 1.37 | 0.933 |
| 3-4 |  |  |  | 0.79 | 0.57, 1.06 | 0.131 |  |  |  | 0.79 | 0.57, 1.07 | 0.152 |
| 5+ |  |  |  | 0.24 | 0.04, 0.74 | **0.042** |  |  |  | 0.23 | 0.04, 0.74 | **0.042** |
| Ever obese before age 30 (BMI) |  |  |  |  |  |  |  |  |  |  |  |  |
| Yes |  |  |  |  |  |  | — | — |  | — | — |  |
| No |  |  |  |  |  |  | 0.22 | 0.21, 0.23 | **<0.001** | 0.22 | 0.21, 0.23 | **<0.001** |
| Unknown |  |  |  |  |  |  | 0.42 | 0.38, 0.45 | **<0.001** | 0.42 | 0.38, 0.45 | **<0.001** |
| ^1^OR = Odds Ratio, CI = Confidence Interval  Model A controls for number of eligible relatives, sex, birth year, non-white race, Hispanic ethnicity, highest parental education.  Model B additionally controls for co-morbidity  Model C excludes comorbidity and adds obesity;  Model D adds co-morbidity and obesity controls. | | | | | | | | | | | | |

Table S6: Relative risk of family history of type 2 diabetes by degree and number of relatives with type 2 diabetes: Interaction Between Sex and Family History of Type 2 Diabetes.

|  | **No Interaction** | | | **With Interaction** | | |
| --- | --- | --- | --- | --- | --- | --- |
| **Characteristic** | **Estimate** | **SE** | **p-value** | **Estimate** | **SE** | **p-value** |
| Sex (reference: Female) | -0.413 |  | **<0.001** | -0.464 |  | **<0.001** |
| Birth year | -0.075 |  | **<0.001** | -0.075 |  | **<0.001** |
| Whether Caucasian |  |  |  |  |  |  |
| Yes | — | — |  | — | — |  |
| No | 0.315 |  | **<0.001** | 0.316 |  | **<0.001** |
| Unknown | -0.252 |  | 0.549 | -0.250 |  | 0.549 |
| Whether Hispanic |  |  |  |  |  |  |
| No | — | — |  | — | — |  |
| Yes | 0.210 |  | **<0.001** | 0.210 |  | **<0.001** |
| Unknown | -1.243 |  | **<0.001** | -1.242 |  | **<0.001** |
| Maximum parental education level |  |  |  |  |  |  |
| HS degree | — | — |  | — | — |  |
| Less than HS | 0.164 |  | **<0.001** | 0.164 |  | **<0.001** |
| Some college | -0.086 |  | **<0.001** | -0.086 |  | **<0.001** |
| College degree | -0.174 |  | **<0.001** | -0.173 |  | **<0.001** |
| Post college | -0.174 |  | **<0.001** | -0.174 |  | **<0.001** |
| Unknown | -0.185 |  | 0.148 | -0.185 |  | 0.150 |
| Number of eligible FDRs | -0.091 |  | **<0.001** | -0.091 |  | **<0.001** |
| Number of eligible FDRs with T2DM |  |  |  |  |  |  |
| 0 | — | — |  | — | — |  |
| 1 | 0.568 |  | **<0.001** | 0.535 |  | **<0.001** |
| 2+ | 1.198 |  | **<0.001** | 1.158 |  | **<0.001** |
| Number of eligible SDRs | -0.007 |  | **0.005** | -0.007 |  | **0.005** |
| Number of eligible SDRs with T2DM |  |  |  |  |  |  |
| 0 | — | — |  | — | — |  |
| 1 | 0.095 |  | **<0.001** | 0.102 |  | **0.004** |
| 2+ | 0.276 |  | **<0.001** | 0.306 |  | **<0.001** |
| Number of eligible FCs | -0.006 |  | **<0.001** | -0.006 |  | **<0.001** |
| Number of eligible FCs with T2DM |  |  |  |  |  |  |
| 0 | — | — |  | — | — |  |
| 1 | 0.067 |  | **0.006** | 0.012 |  | 0.697 |
| 2+ | 0.243 |  | **<0.001** | 0.190 |  | **<0.001** |
| Maximum CCI excluding diabetes before age 30 |  |  |  |  |  |  |
| 0 | — | — |  | — | — |  |
| 1 | 0.045 |  | 0.510 | 0.044 |  | 0.514 |
| 2 | 0.135 |  | 0.281 | 0.136 |  | 0.277 |
| 3-4 | -0.174 |  | 0.154 | -0.176 |  | 0.151 |
| 5+ | -1.154 |  | **0.011** | -1.151 |  | **0.011** |
| Ever obese before age 30 (BMI) |  |  |  |  |  |  |
| Yes | — | — |  | — | — |  |
| No | -1.445 |  | **<0.001** | -1.445 |  | **<0.001** |
| Unknown | -0.768 |  | **<0.001** | -0.769 |  | **<0.001** |
| Male*Number of eligible FDRs with T2DM (1) |  |  |  | 0.078 |  | **0.048** |
| Male *Number of eligible FDRs with T2DM (2+) |  |  |  | 0.095 |  | **0.050** |
| Male *Number of eligible FDRs with T2DM (1) |  |  |  | -0.015 |  | 0.779 |
| Male *Number of eligible FDRs with T2DM (2+) |  |  |  | -0.068 |  | 0.132 |
| Male *Number of eligible FDRs with T2DM (1) |  |  |  | 0.126 |  | **0.008** |
| Male *Number of eligible FDRs with T2DM (2+) |  |  |  | 0.123 |  | **0.019** |
|  |  |  |  |  |  |  |

Estimates refer to regression coefficients, not odds ratios.

Table S7: Relative risk of family history of type 2 diabetes by degree and number of relatives with type 2 diabetes: Hispanic Subsample

|  | **Model A** | | | **Model B** | | | **Model C** | | | **Model D** | | |
| --- | --- | --- | --- | --- | --- | --- | --- | --- | --- | --- | --- | --- |
| **Characteristic** | **OR**^1^ | **95% CI**^1^ | **p-value** | **OR**^1^ | **95% CI**^1^ | **p-value** | **OR**^1^ | **95% CI**^1^ | **p-value** | **OR**^1^ | **95% CI**^1^ | **p-value** |
| Sex | 0.66 | 0.60, 0.73 | **<0.001** | 0.66 | 0.60, 0.73 | **<0.001** | 0.62 | 0.56, 0.68 | **<0.001** | 0.62 | 0.56, 0.68 | **<0.001** |
| Birth year | 0.92 | 0.92, 0.93 | **<0.001** | 0.92 | 0.92, 0.93 | **<0.001** | 0.92 | 0.91, 0.93 | **<0.001** | 0.92 | 0.91, 0.93 | **<0.001** |
| Whether Caucasian |  |  |  |  |  |  |  |  |  |  |  |  |
| Yes | — | — |  | — | — |  | — | — |  | — | — |  |
| No | 1.43 | 1.26, 1.62 | **<0.001** | 1.43 | 1.26, 1.62 | **<0.001** | 1.33 | 1.17, 1.52 | **<0.001** | 1.33 | 1.17, 1.52 | **<0.001** |
| Unknown | 0.00 | 0.00, 0.00 | 0.914 | 0.00 | 0.00, 0.00 | 0.943 | 0.00 | 0.00, 0.00 | 0.915 | 0.00 | 0.00, 0.01 | 0.943 |
| Maximum parental education level |  |  |  |  |  |  |  |  |  |  |  |  |
| HS degree | — | — |  | — | — |  | — | — |  | — | — |  |
| Less than HS | 1.17 | 1.01, 1.35 | **0.032** | 1.17 | 1.01, 1.35 | **0.033** | 1.11 | 0.96, 1.28 | 0.161 | 1.11 | 0.96, 1.28 | 0.163 |
| Some college | 0.87 | 0.77, 0.97 | **0.017** | 0.87 | 0.77, 0.97 | **0.017** | 0.90 | 0.80, 1.02 | 0.098 | 0.90 | 0.80, 1.02 | 0.100 |
| College degree | 0.87 | 0.74, 1.03 | 0.115 | 0.87 | 0.74, 1.03 | 0.115 | 0.94 | 0.79, 1.11 | 0.487 | 0.94 | 0.79, 1.11 | 0.486 |
| Post college | 0.80 | 0.69, 0.93 | **0.003** | 0.80 | 0.69, 0.93 | **0.003** | 0.85 | 0.73, 0.99 | **0.035** | 0.85 | 0.73, 0.99 | **0.037** |
| Unknown | 0.70 | 0.37, 1.19 | 0.216 | 0.70 | 0.37, 1.19 | 0.214 | 0.72 | 0.39, 1.25 | 0.276 | 0.72 | 0.38, 1.25 | 0.275 |
| Number of eligible FDRs | 0.86 | 0.83, 0.89 | **<0.001** | 0.86 | 0.83, 0.89 | **<0.001** | 0.88 | 0.85, 0.91 | **<0.001** | 0.88 | 0.85, 0.91 | **<0.001** |
| Number of eligible FDRs with T2DM |  |  |  |  |  |  |  |  |  |  |  |  |
| 0 | — | — |  | — | — |  | — | — |  | — | — |  |
| 1 | 2.13 | 1.92, 2.37 | **<0.001** | 2.13 | 1.93, 2.37 | **<0.001** | 1.92 | 1.73, 2.13 | **<0.001** | 1.92 | 1.73, 2.13 | **<0.001** |
| 2+ | 4.77 | 4.18, 5.45 | **<0.001** | 4.78 | 4.18, 5.46 | **<0.001** | 3.80 | 3.31, 4.35 | **<0.001** | 3.80 | 3.31, 4.36 | **<0.001** |
| Number of eligible SDRs | 1.00 | 0.99, 1.01 | 0.960 | 1.00 | 0.99, 1.01 | 0.961 | 1.00 | 0.99, 1.01 | 0.895 | 1.00 | 0.99, 1.01 | 0.900 |
| Number of eligible SDRs with T2DM |  |  |  |  |  |  |  |  |  |  |  |  |
| 0 | — | — |  | — | — |  | — | — |  | — | — |  |
| 1 | 1.01 | 0.88, 1.16 | 0.868 | 1.01 | 0.88, 1.17 | 0.858 | 1.02 | 0.88, 1.18 | 0.788 | 1.02 | 0.88, 1.18 | 0.777 |
| 2+ | 1.28 | 1.12, 1.47 | **<0.001** | 1.28 | 1.12, 1.47 | **<0.001** | 1.26 | 1.10, 1.45 | **<0.001** | 1.26 | 1.10, 1.45 | **<0.001** |
| Number of eligible FCs | 0.99 | 0.98, 0.99 | **<0.001** | 0.99 | 0.98, 0.99 | **<0.001** | 0.99 | 0.98, 1.00 | **0.037** | 0.99 | 0.98, 1.00 | **0.037** |
| Number of eligible FCs with T2DM |  |  |  |  |  |  |  |  |  |  |  |  |
| 0 | — | — |  | — | — |  | — | — |  | — | — |  |
| 1 | 1.16 | 1.00, 1.33 | **0.046** | 1.15 | 1.00, 1.33 | **0.048** | 1.08 | 0.94, 1.25 | 0.271 | 1.08 | 0.94, 1.25 | 0.277 |
| 2+ | 1.49 | 1.27, 1.75 | **<0.001** | 1.49 | 1.26, 1.75 | **<0.001** | 1.36 | 1.15, 1.60 | **<0.001** | 1.36 | 1.15, 1.60 | **<0.001** |
| Maximum CCI excluding diabetes before age 30 |  |  |  |  |  |  |  |  |  |  |  |  |
| 0 |  |  |  | — | — |  |  |  |  | — | — |  |
| 1 |  |  |  | 1.13 | 0.78, 1.58 | 0.510 |  |  |  | 1.08 | 0.74, 1.52 | 0.688 |
| 2 |  |  |  | 1.13 | 0.56, 2.03 | 0.711 |  |  |  | 1.09 | 0.54, 2.00 | 0.785 |
| 3-4 |  |  |  | 0.79 | 0.39, 1.43 | 0.476 |  |  |  | 0.83 | 0.41, 1.52 | 0.579 |
| 5+ |  |  |  | 0.00 | 0.00, 0.05 | 0.954 |  |  |  | 0.00 | 0.00, 0.03 | 0.952 |
| Ever obese before age 30 (BMI) |  |  |  |  |  |  |  |  |  |  |  |  |
| Yes |  |  |  |  |  |  | — | — |  | — | — |  |
| No |  |  |  |  |  |  | 0.24 | 0.21, 0.26 | **<0.001** | 0.24 | 0.21, 0.26 | **<0.001** |
| Unknown |  |  |  |  |  |  | 0.46 | 0.40, 0.54 | **<0.001** | 0.46 | 0.40, 0.54 | **<0.001** |
| ^1^OR = Odds Ratio, CI = Confidence Interval  Model A controls for number of eligible relatives, sex, birth year, non-white race, Hispanic ethnicity, highest parental education.  Model B additionally controls for co-morbidity  Model C excludes comorbidity and adds obesity;  Model D adds co-morbidity and obesity controls. | | | | | | | | | | | | |

Table S8: Relative risk of family history of type 2 diabetes by degree and number of relatives with type 2 diabetes Non-Hispanics Subsample

|  | **Model A** | | | **Model B** | | | **Model C** | | | **Model D** | | |
| --- | --- | --- | --- | --- | --- | --- | --- | --- | --- | --- | --- | --- |
| **Characteristic** | **OR**^1^ | **95% CI**^1^ | **p-value** | **OR**^1^ | **95% CI**^1^ | **p-value** | **OR**^1^ | **95% CI**^1^ | **p-value** | **OR**^1^ | **95% CI**^1^ | **p-value** |
| Sex | 0.72 | 0.69, 0.74 | **<0.001** | 0.72 | 0.69, 0.74 | **<0.001** | 0.67 | 0.64, 0.69 | **<0.001** | 0.67 | 0.64, 0.69 | **<0.001** |
| Birth year | 0.93 | 0.93, 0.94 | **<0.001** | 0.93 | 0.93, 0.94 | **<0.001** | 0.93 | 0.92, 0.93 | **<0.001** | 0.93 | 0.93, 0.93 | **<0.001** |
| Whether Caucasian |  |  |  |  |  |  |  |  |  |  |  |  |
| Yes | — | — |  | — | — |  | — | — |  | — | — |  |
| No | 1.61 | 1.49, 1.73 | **<0.001** | 1.61 | 1.49, 1.73 | **<0.001** | 1.38 | 1.28, 1.49 | **<0.001** | 1.38 | 1.28, 1.49 | **<0.001** |
| Unknown | 0.00 |  | 0.851 | 0.00 |  | 0.851 | 0.00 |  | 0.855 | 0.00 |  | 0.855 |
| Maximum parental education level |  |  |  |  |  |  |  |  |  |  |  |  |
| HS degree | — | — |  | — | — |  | — | — |  | — | — |  |
| Less than HS | 1.24 | 1.12, 1.38 | **<0.001** | 1.24 | 1.12, 1.38 | **<0.001** | 1.20 | 1.08, 1.33 | **<0.001** | 1.20 | 1.08, 1.33 | **<0.001** |
| Some college | 0.89 | 0.85, 0.93 | **<0.001** | 0.89 | 0.85, 0.93 | **<0.001** | 0.92 | 0.88, 0.97 | **<0.001** | 0.92 | 0.88, 0.97 | **<0.001** |
| College degree | 0.76 | 0.71, 0.80 | **<0.001** | 0.76 | 0.71, 0.80 | **<0.001** | 0.83 | 0.78, 0.89 | **<0.001** | 0.83 | 0.78, 0.88 | **<0.001** |
| Post college | 0.74 | 0.70, 0.79 | **<0.001** | 0.74 | 0.70, 0.79 | **<0.001** | 0.84 | 0.79, 0.89 | **<0.001** | 0.83 | 0.79, 0.88 | **<0.001** |
| Unknown | 0.82 | 0.61, 1.07 | 0.163 | 0.82 | 0.61, 1.07 | 0.164 | 0.86 | 0.64, 1.13 | 0.301 | 0.86 | 0.64, 1.13 | 0.301 |
| Number of eligible FDRs | 0.90 | 0.89, 0.91 | **<0.001** | 0.90 | 0.89, 0.91 | **<0.001** | 0.92 | 0.91, 0.93 | **<0.001** | 0.92 | 0.91, 0.93 | **<0.001** |
| Number of eligible FDRs with T2DM |  |  |  |  |  |  |  |  |  |  |  |  |
| 0 | — | — |  | — | — |  | — | — |  | — | — |  |
| 1 | 1.96 | 1.88, 2.04 | **<0.001** | 1.96 | 1.88, 2.04 | **<0.001** | 1.75 | 1.67, 1.82 | **<0.001** | 1.75 | 1.67, 1.82 | **<0.001** |
| 2+ | 4.13 | 3.92, 4.35 | **<0.001** | 4.13 | 3.92, 4.36 | **<0.001** | 3.24 | 3.07, 3.41 | **<0.001** | 3.24 | 3.07, 3.42 | **<0.001** |
| Number of eligible SDRs | 0.99 | 0.99, 1.00 | **0.003** | 0.99 | 0.99, 1.00 | **0.003** | 0.99 | 0.99, 1.00 | **0.003** | 0.99 | 0.99, 1.00 | **0.003** |
| Number of eligible SDRs with T2DM |  |  |  |  |  |  |  |  |  |  |  |  |
| 0 | — | — |  | — | — |  | — | — |  | — | — |  |
| 1 | 1.11 | 1.04, 1.17 | **<0.001** | 1.11 | 1.04, 1.17 | **<0.001** | 1.10 | 1.04, 1.17 | **0.001** | 1.10 | 1.04, 1.17 | **0.001** |
| 2+ | 1.36 | 1.29, 1.44 | **<0.001** | 1.36 | 1.29, 1.44 | **<0.001** | 1.31 | 1.24, 1.39 | **<0.001** | 1.31 | 1.24, 1.39 | **<0.001** |
| Number of eligible FCs | 0.99 | 0.99, 0.99 | **<0.001** | 0.99 | 0.99, 0.99 | **<0.001** | 0.99 | 0.99, 1.00 | **<0.001** | 0.99 | 0.99, 1.00 | **<0.001** |
| Number of eligible FCs with T2DM |  |  |  |  |  |  |  |  |  |  |  |  |
| 0 | — | — |  | — | — |  | — | — |  | — | — |  |
| 1 | 1.09 | 1.04, 1.15 | **<0.001** | 1.09 | 1.04, 1.15 | **<0.001** | 1.07 | 1.02, 1.13 | **0.010** | 1.07 | 1.02, 1.13 | **0.010** |
| 2+ | 1.34 | 1.26, 1.42 | **<0.001** | 1.34 | 1.26, 1.42 | **<0.001** | 1.28 | 1.20, 1.36 | **<0.001** | 1.28 | 1.20, 1.35 | **<0.001** |
| Maximum CCI excluding diabetes before age 30 |  |  |  |  |  |  |  |  |  |  |  |  |
| 0 |  |  |  | — | — |  |  |  |  | — | — |  |
| 1 |  |  |  | 1.06 | 0.92, 1.22 | 0.395 |  |  |  | 1.05 | 0.91, 1.22 | 0.473 |
| 2 |  |  |  | 1.11 | 0.84, 1.44 | 0.439 |  |  |  | 1.13 | 0.85, 1.47 | 0.366 |
| 3-4 |  |  |  | 0.84 | 0.64, 1.08 | 0.192 |  |  |  | 0.83 | 0.63, 1.07 | 0.160 |
| 5+ |  |  |  | 0.31 | 0.10, 0.74 | **0.021** |  |  |  | 0.30 | 0.09, 0.71 | **0.017** |
| Ever obese before age 30 (BMI) |  |  |  |  |  |  |  |  |  |  |  |  |
| Yes |  |  |  |  |  |  | — | — |  | — | — |  |
| No |  |  |  |  |  |  | 0.24 | 0.23, 0.25 | **<0.001** | 0.24 | 0.23, 0.25 | **<0.001** |
| Unknown |  |  |  |  |  |  | 0.46 | 0.43, 0.49 | **<0.001** | 0.46 | 0.43, 0.49 | **<0.001** |
| ^1^OR = Odds Ratio, CI = Confidence Interval  Model A controls for number of eligible relatives, sex, birth year, non-white race, Hispanic ethnicity, highest parental education.  Model B additionally controls for co-morbidity  Model C excludes comorbidity and adds obesity;  Model D adds co-morbidity and obesity controls. | | | | | | | | | | | | |

Table S9: Relative risk of family history of type 2 diabetes by degree and number of relatives with type 2 diabetes: Interaction Between Hispanic Ethnicity and Family History of Type 2 Diabetes.

|  | **No Interaction** | | | **With Interaction** | | |
| --- | --- | --- | --- | --- | --- | --- |
| **Characteristic** | **Estimate** | **SE** | **p-value** | **Estimate** | **SE** | **p-value** |
| Sex | -0.416 | 0.018 | **<0.001** | -0.417 | 0.018 | **<0.001** |
| Birth year | -0.076 | 0.002 | **<0.001** | -0.076 | 0.002 | **<0.001** |
| Whether Caucasian |  |  |  |  |  |  |
| Yes | — | — |  | — | — |  |
| No | 0.318 | 0.034 | **<0.001** | 0.319 | 0.034 | **<0.001** |
| Unknown | -10.213 | 50.264 | 0.839 | -10.198 | 50.226 | 0.839 |
| Whether Hispanic |  |  |  |  |  |  |
| No | — | — |  | — | — |  |
| Yes | 0.210 | 0.026 | **<0.001** | 0.152 | 0.054 | **0.005** |
| Maximum parental education level |  |  |  |  |  |  |
| HS degree | — | — |  | — | — |  |
| Less than HS | 0.161 | 0.043 | **<0.001** | 0.161 | 0.043 | **<0.001** |
| Some college | -0.085 | 0.023 | **<0.001** | -0.084 | 0.023 | **<0.001** |
| College degree | -0.171 | 0.029 | **<0.001** | -0.171 | 0.029 | **<0.001** |
| Post college | -0.176 | 0.028 | **<0.001** | -0.176 | 0.028 | **<0.001** |
| Unknown | -0.181 | 0.130 | 0.164 | -0.178 | 0.130 | 0.170 |
| Number of eligible FDRs | -0.092 | 0.005 | **<0.001** | -0.092 | 0.006 | **<0.001** |
| Number of eligible FDRs with T2DM |  |  |  |  |  |  |
| 0 | — | — |  | — | — |  |
| 1 | 0.569 | 0.020 | **<0.001** | 0.557 | 0.021 | **<0.001** |
| 2+ | 1.195 | 0.025 | **<0.001** | 1.178 | 0.027 | **<0.001** |
| Number of eligible SDRs | -0.007 | 0.002 | **0.006** | -0.007 | 0.002 | **0.006** |
| Number of eligible SDRs with T2DM |  |  |  |  |  |  |
| 0 | — | — |  | — | — |  |
| 1 | 0.087 | 0.027 | **0.001** | 0.095 | 0.030 | **0.001** |
| 2+ | 0.268 | 0.026 | **<0.001** | 0.270 | 0.028 | **<0.001** |
| Number of eligible FCs | -0.007 | 0.001 | **<0.001** | -0.007 | 0.001 | **<0.001** |
| Number of eligible FCs with T2DM |  |  |  |  |  |  |
| 0 | — | — |  | — | — |  |
| 1 | 0.069 | 0.025 | **0.005** | 0.066 | 0.026 | **0.011** |
| 2+ | 0.251 | 0.029 | **<0.001** | 0.241 | 0.031 | **<0.001** |
| Maximum CCI excluding diabetes before age 30 |  |  |  |  |  |  |
| 0 | — | — |  | — | — |  |
| 1 | 0.055 | 0.069 | 0.425 | 0.055 | 0.069 | 0.424 |
| 2 | 0.121 | 0.128 | 0.342 | 0.120 | 0.128 | 0.350 |
| 3-4 | -0.190 | 0.125 | 0.127 | -0.190 | 0.125 | 0.127 |
| 5+ | -1.355 | 0.506 | **0.007** | -1.355 | 0.506 | **0.007** |
| Ever obese before age 30 (BMI) |  |  |  |  |  |  |
| Yes | — | — |  | — | — |  |
| No | -1.427 | 0.020 | **<0.001** | -1.427 | 0.020 | **<0.001** |
| Unknown | -0.768 | 0.030 | **<0.001** | -0.769 | 0.030 | **<0.001** |
| Hispanic*Number of eligible FDRs with T2DM (1) |  |  |  | 0.087 | 0.057 | 0.128 |
| Hispanic*Number of eligible FDRs with T2DM (2+) |  |  |  | 0.123 | 0.070 | ***0.081*** |
| Hispanic*Number of eligible FDRs with T2DM (1) |  |  |  | -0.054 | 0.076 | 0.474 |
| Hispanic*Number of eligible FDRs with T2DM (2+) |  |  |  | -0.012 | 0.064 | 0.856 |
| Hispanic*Number of eligible FDRs with T2DM (1) |  |  |  | 0.019 | 0.075 | 0.804 |
| Hispanic*Number of eligible FDRs with T2DM (2+) |  |  |  | 0.069 | 0.078 | 0.377 |
|  |  |  |  |  |  |  |

Note: We excluded individuals with unknown ethnicity.

Estimates refer to regression coefficients, not odds ratios.
